# Loss of JAK1 Drives Innate Immune Deficiency

**DOI:** 10.3389/fimmu.2018.03108

**Published:** 2019-01-08

**Authors:** Agnieszka Witalisz-Siepracka, Klara Klein, Daniela Prinz, Nicoletta Leidenfrost, Gernot Schabbauer, Alexander Dohnal, Veronika Sexl

**Affiliations:** ^1^Institute of Pharmacology and Toxicology, University of Veterinary Medicine, Vienna, Austria; ^2^Center for Physiology and Pharmacology, Institute for Physiology, Medical University of Vienna, Vienna, Austria; ^3^Tumor Immunology, St. Anna Kinderkrebsforschung, Children's Cancer Research Institute, Vienna, Austria

**Keywords:** JAK-STAT, natural killer cells, JAK1, JAK2, tumor surveillance

## Abstract

The Janus kinase—signal transducers and activators of transcription (JAK-STAT) signaling pathway is critical in tuning immune responses and its dysregulation is tightly associated with cancer and immune disorders. Disruption of interleukin (IL)-15/STAT5 signaling pathway due to the loss of IL-15 receptor chains, JAK3 or STAT5 leads to immune deficiencies with natural killer (NK) cell abnormalities. JAK1, together with JAK3 transmits signals downstream of IL-15, but the exact contribution of JAK1 to NK cell biology remains to be elucidated. To study the consequences of JAK1 deficiency in NK cells, we generated mice with conditional deletion of JAK1 in NKp46^+^ cells (*Jak1*^*fl*/*fl*^*Ncr1Cre*). We show here that deletion of NK cell-intrinsic JAK1 significantly reduced NK cell numbers in the bone marrow and impaired their development. In line, we observed almost a complete loss of NK cells in the spleen, blood, and liver, proving a crucial role of JAK1 in peripheral NK cells. In line, *Jak1*^*fl*/+^*Ncr1Cre* mice showed significantly impaired NK cell-mediated tumor surveillance. Our data suggest that JAK2 is not able to compensate for the loss of JAK1 in NK cells. Importantly, conditional deletion of JAK2 in NKp46^+^ cells had no effect on peripheral NK cells revealing that NK cell-intrinsic JAK2 is dispensable for NK cell survival. In summary, we identified that loss of JAK1 in NK cells drives innate immune deficiency, whereas JAK2 deficiency leaves NK cell numbers and maturation unaltered. We thus propose that in contrast to currently used JAK1/JAK2 inhibitors, the use of JAK2-specific inhibitors would be advantageous for the patients by leaving NK cells intact.

## Introduction

Natural killer (NK) cells are innate lymphocytes which recognize and kill virally infected or transformed cells (1). Deficiency of NK cells is a rare but increasingly appreciated subtype of primary immunodeficiency (PID). Classical NK cell deficiency is characterized by the absence of NK cells in the peripheral blood and results in enhanced susceptibility to viral infections (2).

The Janus kinase (JAK)—signal transducer and activator of transcription (STAT) signaling pathway acts downstream of multiple cytokines, growth factors, and hormones thereby critically regulating immune responses (3, 4). Upon binding of a specific ligand to its cognate receptor, conformational changes lead to receptor oligomerization and activation of the receptor-associated JAKs. JAKs auto- and trans-phosphorylate one another and phosphorylate receptor chains, providing the docking sites for STAT molecules. STATs then undergo JAK-mediated phosphorylation, dimerize, and translocate to the nucleus, where they regulate the transcription of target genes (5). JAK3 and STAT5 are crucial players in transducing the signal downstream of cytokines which utilize γc receptor (6). “Loss-of-function” (LOF) mutations in genes encoding JAK3 (7) or STAT5B (8) lead to PIDs with an NK cell abnormality underlining the importance of the pathway for innate lymphocytes. The immunodeficiency of these patients has been explained by impaired IL-7 and IL-15 responses (6). Importantly, JAK1 has a dominant role over JAK3 in activating STAT5 downstream of γc-containing cytokine receptors (9). It is attractive to speculate that LOF mutations of JAK1 could also result in PID. To date, only one patient harboring JAK1 germline mutations, where JAK1 was reduced but not absent, has been identified and indeed presented with immune suppression (10).

In mice, complete loss of JAK1 leads to perinatal lethality and newborn mice display a strong reduction of thymocytes and B cells (11). These observations were confirmed in adult mice: inducible deletion of JAK1 leads to impairment of hematopoietic stem cells (HSCs) homeostasis and markedly reduces the frequencies of B cells and the B220^+^CD11c^+^NK1.1^+^ subset of NK cells (12). However, to date, no study has directly analyzed the effect of loss of JAK1 on conventional NK cells.

The first insights into the contribution of JAK1 to NK cell biology derive from studies using JAK inhibitors—approved drugs for treatment of cancers and autoimmune diseases (13). Both, mice and patients treated with the JAK1/JAK2 inhibitor Ruxolitinib showed reduced NK cell numbers, impaired maturation, and function (14, 15). Since JAK2 has also been implicated in driving NK cell differentiation (14, 16), it remains to be elucidated which of the two kinases is responsible for the observed effects of Ruxolitinib treatment.

Using mice with knockout of *Jak1* or *Jak2* in NKp46^+^ cells, we show here that JAK2 is dispensable for NK cell survival. In contrast, deletion of JAK1 in mature NK cells leads to NK cell deficiency and loss of one allele of *Jak1* is sufficient to impair tumor growth control. Thus, we identified JAK1 as a key factor for mature NK cells and generated a mouse model of classical NK cell deficiency.

## Materials and Methods

### Mice and Cell Lines

*Jak1*^*fl*/*fl*^ (*C57BL/6N-Jak1*^*tm*1*c*(*EUCOMM*)*Hmgu*/*H*^; were kindly provided by Dr. Alexander Dohnal (CCRI, Vienna, Austria). The *Jak1*^*tm*1*c*^ allele of the mutant was generated from mice with the *Jak1*^*tm*1*a*^ knockout first allele (described by International Mouse Phenotyping Consortium https://www.mousephenotype.org) by excision of the lacZ-neo cassette via Flp-recombination. The conditional potential of *Jak1*^*fl*/*fl*^ mice was activated by Cre-recombination and excision of the loxP-flanked exon 3 of *Jak1*. Tissue-specific recombination was induced by cross breeding of *Jak1*^*fl*/*fl*^ or *Jak2*^*fl*/*fl*^ [*Jak2*^*tm*1*Kuw*^; (17)] with B6N-Tg(Ncr1Cre); (18) mice. *Stat5*^*fl*/*fl*^ (19) and *Stat5*^*fl*/*fl*^*Ncr1Cre* (18) mice were described before. *Jak1*^*fl*/*fl*^*, Ncr1Cre, Stat5*^*fl*/*fl*^*, Stat5*^*fl*/*fl*^*Ncr1Cre* mice were on C57B6/N background and *Jak2*^*fl*/*fl*^ were on mixed background. The experimental animals were age-matched (8–12 weeks) and maintained under specific pathogen-free conditions at the University of Veterinary Medicine, Vienna according to Federation for Laboratory Animal Science Associations (FELASA) guidelines (2014). The animal experiments were approved by the Ethics and Animal Welfare Committee of the University of Veterinary Medicine Vienna and the national authority (Austrian Federal Ministry of Science and Research) according to §§ 26ff. of Animal Experiments Act, Tierversuchsgesetz 2012—TVG 2012, under licenses BMWF-68.205/0218-II/3b/2012 and BMBWF-68.205/0174-V/3b/2018 and were conducted according to the guidelines of FELASA and ARRIVE. Throughout the paper *Jak1*^*WT*^ refers to pooled data from *Jak1*^*fl*/+^ and *Jak1*^*fl*/*fl*^ mice.

The mouse lymphoma cell lines RMA-Rae1 [kindly provided by Prof. A. Cerwenka; (20)] and YAC-1 were cultured in RPMI1640 (Sigma) complete medium containing 10% FCS (Bio & Sell), 100 U/mL penicillin, 100 mg/mL streptomycin (Sigma), and 50 μM 2-mercaptoethanol (Sigma).

### *In vivo* Tumor Model

*Jak1*^*fl*/+^ and *Jak1*^*fl*/+^*Ncr1Cre* mice were injected *s.c*. with 10^6^ RMA-Rae1 cells into both flanks and the tumor growth was monitored every other day. Ten days post injection the mice were sacrificed and the tumor weight was determined. For flow cytometric analysis of tumor infiltrating NK cells, tumors were cut into ~5 mm^2^ pieces and the single cell suspension was obtained using gentleMACS™ *Octo Dissociator* (Miltenyi Biotec) with digestion buffer containing Collagenase D (1 mg/mL; Sigma Aldrich) and DNAse I (20 mg/mL; Roche).

### NK-Cell Isolation, Expansion, and Stimulation

NK cells were isolated from spleen single-cell suspensions using DX5-labeled MACS beads according to the manufacturer's instructions (Miltenyi Biotec). NK cells were expanded in RPMI1640 complete medium supplemented with 5,000 U/mL rhIL-2 (Proleukin, Novartis) for 7 days. The number of CD3^−^NK1.1^+^ cells was assessed by flow cytometry on day 0, 3, 5, and 7. On day 7 cells were lysed for Western blot analysis. For pSTAT5 analysis 10^6^ splenocytes were stimulated with 50 ng/ml rmIL-15 (PeproTech) for 15 min and the cells were fixed in 2% PFA followed by methanol permeabilization and rehydration.

### NK-Cell Cytotoxicity Assay

For *in vitro* cytotoxicity assays, DX5-MACS–sorted NK cells were expanded for 7 days in IL-2 as described above and mixed at indicated effector: target ratios with carboxyfluorescein diacetate succinimidyl ester (CFSE, Molecular Probes, CellTrace CFSE Cell Proliferation Kit) labeled target cells. After 4 h of incubation at 37°C, the cells were stained with Sytox Blue Dead Cell Stain (Thermo Fischer) and the specific target cell lysis was assessed by flow cytometry.

### Flow Cytometry

Single cell suspensions were prepared from spleen, bone marrow, or liver. Liver was perfused via the portal vein with 5–10 mL sterile PBS. Separation of lymphocytes was performed using 37.5% percoll (GE Healthcare). For blood analysis, the erythrocytes were lysed using BD FACS Lysing Solution according to manufacturer's protocol (BD Bioscience). The antibodies (clones) targeting following proteins were purchased from eBioscience: CD3 (17A2), CD3e (145-2C11), CD11b (M1/70), CD16/CD32 (93), CD19 (eBio1D3) CD27 (LG.7F9), CD49b (DX5), CD122 (5H4), CD226 (10E5), Gr-1 (RB6-8C5), KLRG1 (2F1), Ly49A (A1), Ly49G2 (eBio4D11), NKG2A/C/E (20d5), NKG2D (CX5), NKp46 (29A1.4), NK1.1 (PK136), and Ter119 (TER-119). CD49a (Ha31/8) and pSTAT5 [47/Stat5(pY694)] antibodies were purchased from BD Pharminogen and pan-Rae1 (186107) was purchased from R&D Systems. Total cell numbers were assessed by flow cytometry using counting beads Count Bright Beads (Invitrogen). Flow cytometry experiments were performed on a BD FACSCanto II (BD Bioscience) or Cytoflex (Beckman Coulter) and analyzed using BD FACSDiva V8.0 (BD Bioscience), CytExpert (Beckman Coulter) or FlowJo V10 (FlowJo, LLC) software.

### Western Blot

Cell lysis, SDS-PAGE, and Western blots were performed as described previously (21). The detection of chemiluminescence was performed using Clarity Western ECL substrate (BioRad) and the ChemiDocT XRS+ Molecular Imager (BioRad) and analyzed by Image Lab software (BioRad). The following antibodies were used: anti–β-actin (C4, sc-47778) from Santa Cruz as loading control, anti-JAK2 (D2E12; #3230), anti-Perforin (#3693) and anti-JAK1 (#3332) from Cell Signaling Technology.

### Statistical Analysis

Unpaired *t*-tests or one-way ANOVA with Tukey post tests were performed using GraphPad Prism version 5.00 (GraphPad Software). The level of significance is indicated for each experiment (^*^p < 0.05; ^**^p < 0.01; ^***^p < 0.001).

## Results

### JAK1 Deletion Reduces NK Cell and ILC1 Numbers in a Dose-Dependent Manner

**Figure 1:**
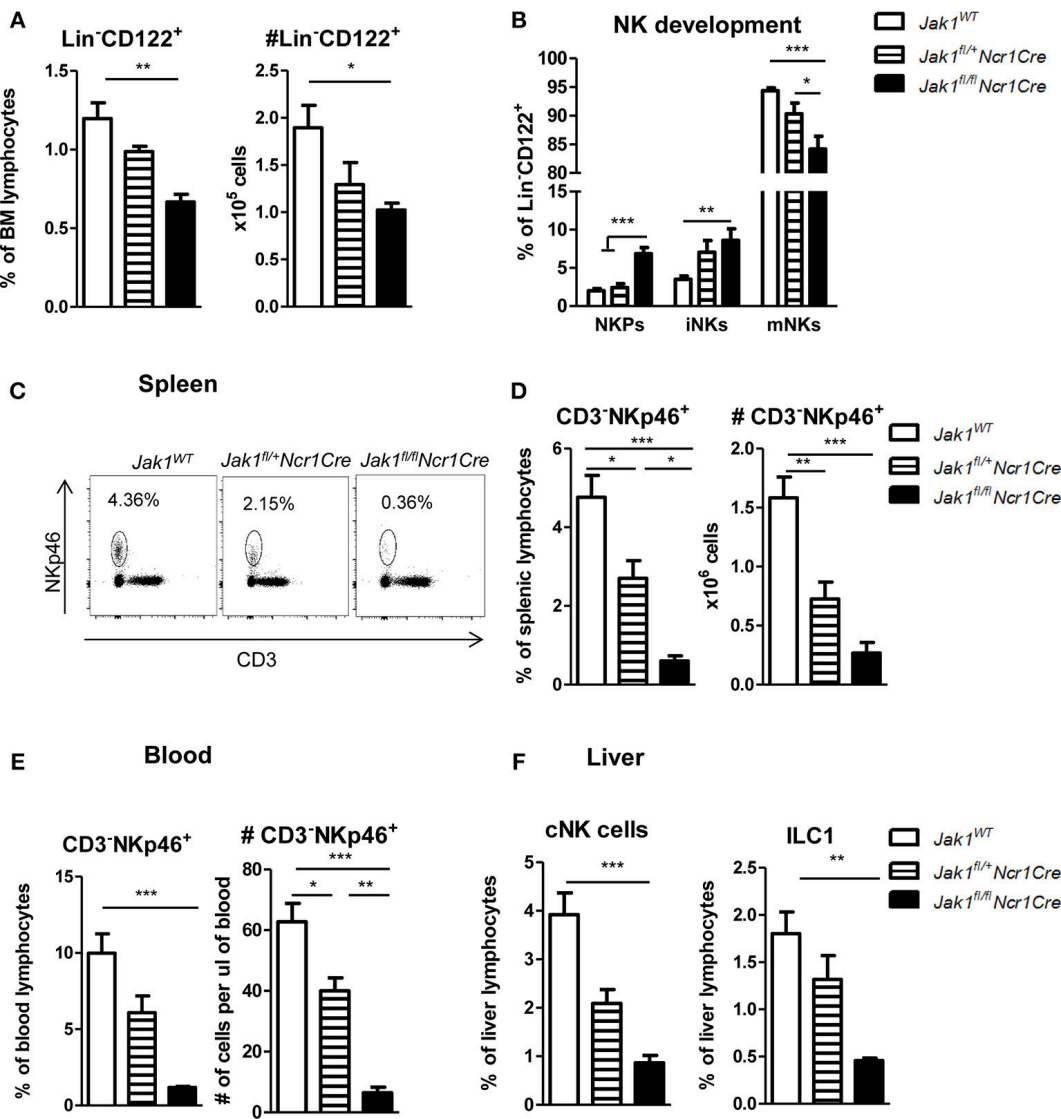
Loss of JAK1 in NKp46^+^ cells leads to an almost complete absence of peripheral NK cells. **(A)** Frequency (left panel) and total numbers (right panel) of Lin^−^(CD3^−^CD19^−^Ly-6G^−^Ter119^−^) CD122^+^ NK cells in the bone marrow were assessed by flow cytometry. **(B)** Bone marrow Lin^−^CD122^+^ cells were further divided into NK precursors (NKPs: NKp46^−^NK1.1^−^), immature NK cells (iNKs: NKp46^−^NK1.1^+^), and mature NK cells (mNKs: NKp46^+^NK1.1^+^). **(C)** Frequency of CD3^−^NKp46^+^ NK cells in the spleen was assessed by flow cytometry and representative plots are shown. **(D,E)** Frequency (left panel) and total numbers (right panel) of CD3^−^NKp46^+^ NK cells in the **(D)** spleen and **(E)** blood were assessed by flow cytometry. **(F)** Frequency of conventional NK cells (CD3^−^NK1.1^+^NKp46^+^CD49b^+^, left panel) and ILC1 cells (CD3^−^NK1.1^+^NKp46^+^CD49a^+^, right panel) was analyzed in the liver of *Jak1*^*WT*^, *Jak1*^*fl*/+^*Ncr1Cre*, and *Jak1*^*fl*/*fl*^*Ncr1Cre* mice by flow cytometry. **(A,B,D–F)** Bar graphs represent mean ± SEM of 1–2 independent experiments; *n* = 3–11. *p < 0.05, **p < 0.01, ***p < 0.001.

The JAK1/JAK2 inhibitor Ruxolitinib has been shown to reduce NK cell numbers, maturation, and function (14, 15). To compare and address the contribution of JAK1 and JAK2 for NK cell biology we generated mice with conditional deletion of either JAK1 or JAK2 in NKp46^+^ cells. We thus crossed *Ncr1Cre* (18) mice with *Jak1*^*fl*/*fl*^ or *Jak2*^*fl*/*fl*^ [see Materials and Methods section and (17)] mice, respectively. NK cells develop in the bone marrow from NK cell precursors (NKPs), which are defined as Lin^−^CD122^+^NK1.1^−^NKp46^−^. They develop into immature NK cells (iNKs) that become NK1.1^+^ while only mature NK cells (mNKs) are NK1.1^+^NKp46^+^ (22). As the *Cre* recombinase expression in *Ncr1Cre* mice is driven by the NKp46 promoter, *Cre*-mediated deletion is restricted to mNK cells. We observed a significant decrease of percentage and total numbers of bone marrow NK cells in *Jak1*^*fl*/*fl*^*Ncr1Cre* mice (Figure 1A). The Lin^−^CD122^+^ NK cells in *Jak1*^*fl*/*fl*^*Ncr1Cre* mice showed enriched percentages of NK cell precursors (NKPs) and immature NK cells, while mNKs were significantly reduced in the bone marrow in line with a developmental block at the iNK cell stage preventing progression to mNK cell stage (Figure 1B). Deletion of one allele of *Jak1* led to intermediate numbers of bone marrow NK cells (Figure 1A). Consistently, NK cell development showed an intermediate phenotype suggesting a *Jak1* gene-dosage effect on NK cell development (Figure 1B).

The block in development of bone marrow NK cells translated into drastically reduced numbers of NK cells in the periphery. Loss of JAK1 led to an almost complete deficiency of splenic and blood NK cells (Figures 1C–E). In line with a *Jak1* gene dosage effect, *Jak1*^*fl*/+^*Ncr1Cre* mice displayed reduced NK cell percentages and total numbers to 50% compared to wild-type littermates in the spleen and blood (Figures 1C–E). Deletion of the JAK1 downstream effector and transcription factor STAT5 in NKp46^+^ cells also leads to a reduction of mature NK cells (18). A direct comparison of *Jak1*^*fl*/*fl*^*Ncr1Cre* and *Stat5*^*fl*/*fl*^*Ncr1Cre* mice revealed that deletion of JAK1 provoked an even more pronounced NK cell deficiency in spleen and blood than deletion of STAT5 (Figures S1A,B).

Liver NKp46^+^ innate lymphocytes comprise two groups of distinct lineages (23). Conventional NK cells (cNK) are characterized by expression of CD49b and circulate freely whereas liver resident type 1 innate lymphocytes (ILC1) are characterized by the expression of CD49a and are restricted to the liver (24). Similarly to spleen and blood, liver cNKs and tissue resident ILC1s were almost completely ablated upon loss of JAK1 (Figure 1F). Again the deletion of one allele of *Jak1* resulted in an intermediate abundance of liver innate lymphocytes (Figure 1F). In summary, these findings led us to conclude that JAK1 expression in NKp46^+^ cells is indispensable for NK cell development and maintenance in peripheral organs in a dose-dependent manner.

### JAK1 Is Crucial for NK Cell Maturation

**Figure 2:**
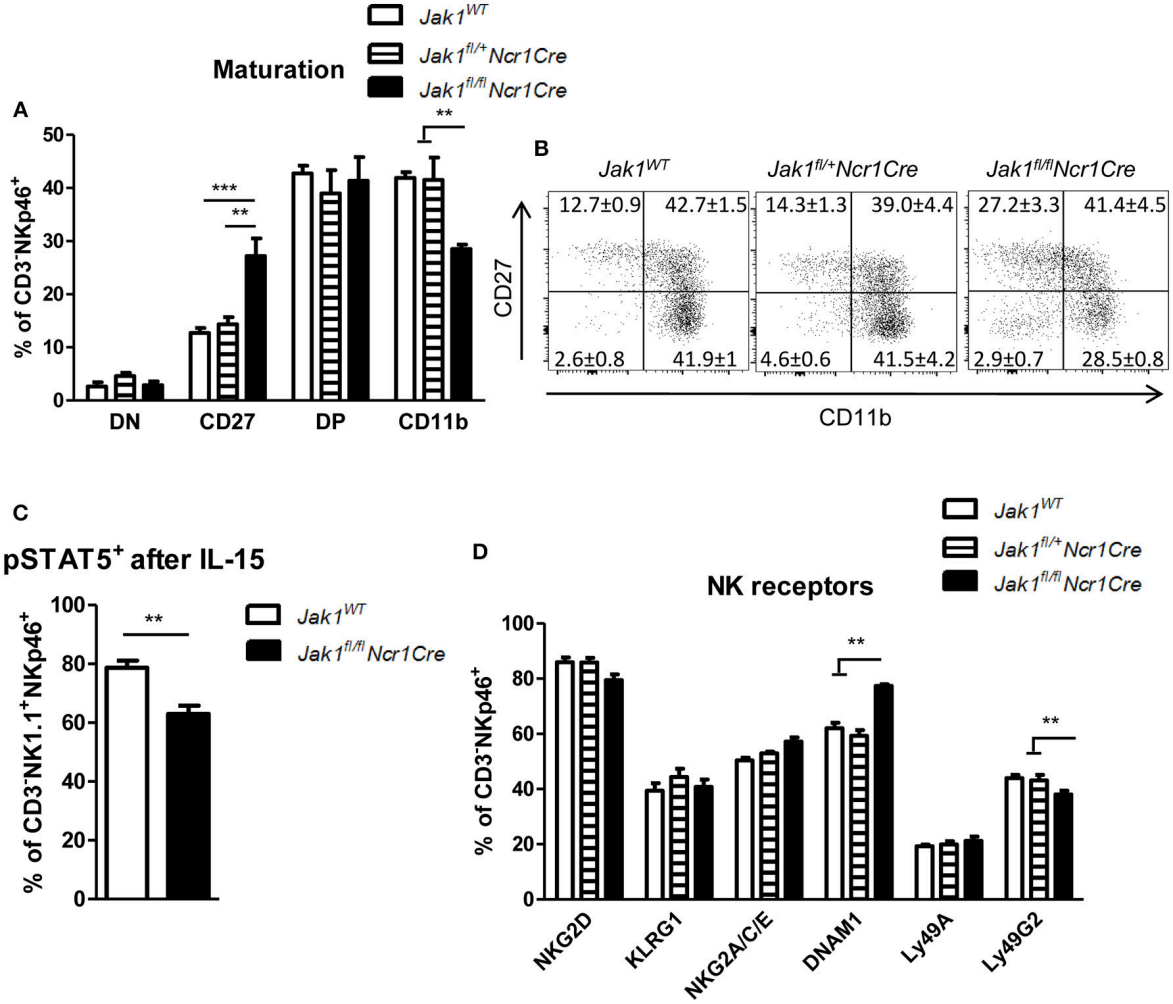
The remaining *Jak1*^*fl*/*fl*^*Ncr1Cre* NK cells show an immature phenotype. **(A,B)** Splenic CD3^−^NKp46^+^ NK cells were analyzed for expression of CD27 and CD11b by flow cytometry. **(A)** Frequency of cells in each maturation stage is shown: DN (CD27^−^CD11b^−^), CD27 (CD27^+^CD11b^−^), DP (CD27^+^CD11b^+^), CD11b (CD27^−^CD11b^+^). The total numbers of cells in each maturation stage are shown in Figure S1C. **(B)** Representative plots are shown. **(C)** pSTAT5(Y694)^+^ cells were analyzed within the CD3^−^NKp46^+^NK1.1^+^ population *ex vivo* after 15 min stimulation with IL-15 by flow cytometry. **(D)** Splenic CD3^−^NKp46^+^ NK cells were analyzed for expression of the indicated activating and inhibitory receptors by flow cytometry. The percentage of NK cells positive for each receptor is shown. The median fluorescence intensity data is presented in Figure S1D. **(A–D)** Bar graphs and numbers on the plots represent mean ± SEM of 2 independent experiments; *n* = 5–8. **p < 0.01, ***p < 0.001.

In the periphery NK cells undergo maturation steps which are characterized by sequential expression of CD27 and CD11b surface markers (22). One allele of *Jak1* was sufficient to drive NK cell maturation as we did not detect any differences in percentage of cells in each maturation stage between *Jak1*^*WT*^ and *Jak1*^*fl*/+^*Ncr1Cre* cells (Figures 2A,B). The remaining *Jak1*^*fl*/*fl*^*Ncr1Cre* NK cells showed an increase in the immature population (CD27^+^CD11b^−^) and a decrease in the mature CD27^−^CD11b^+^ population (Figures 2A,B and Figure S1C). This result suggests that the remaining cells might just have lost JAK1 and have not received sufficient IL-15 signaling to fully mature. Indeed, the remaining *Jak1*^*fl*/*fl*^*Ncr1Cre* NK cells showed reduced phosphorylation of STAT5 *ex vivo* upon short-term stimulation with IL-15 (Figure 2C). NK cell activity is controlled by a balance between activating and inhibitory receptors. Deletion of neither one nor both alleles of *Jak1* had an effect on the percentage of NK cells expressing following activating and inhibitory receptors: KLRG1, NKG2D, NKG2A/C/E, and Ly49A (Figure 2D). The most prominent difference was a slight decrease of Ly49G2^+^ NK cells and an increase in DNAM-1^+^ NK cells in the *Jak1*^*fl*/*fl*^*Ncr1Cre* mice (Figure 2D). Similarly, no gross differences were detected in the expression level (MFI) of each receptor, besides an increase in the MFI of DNAM1 and Ly49G2 (Figure S1D). Besides its role as an activating receptor, DNAM-1 expression marks a developmental step; DNAM-1^+^ cells give rise to DNAM-1^−^ cells (25). We reasoned that the changes in DNAM-1^+^ reflect the maturation block of *Jak1*^*fl*/*fl*^*Ncr1Cre* NK cells.

### JAK2 Is Dispensable for NK Cell Survival and Maturation

**Figure 3:**
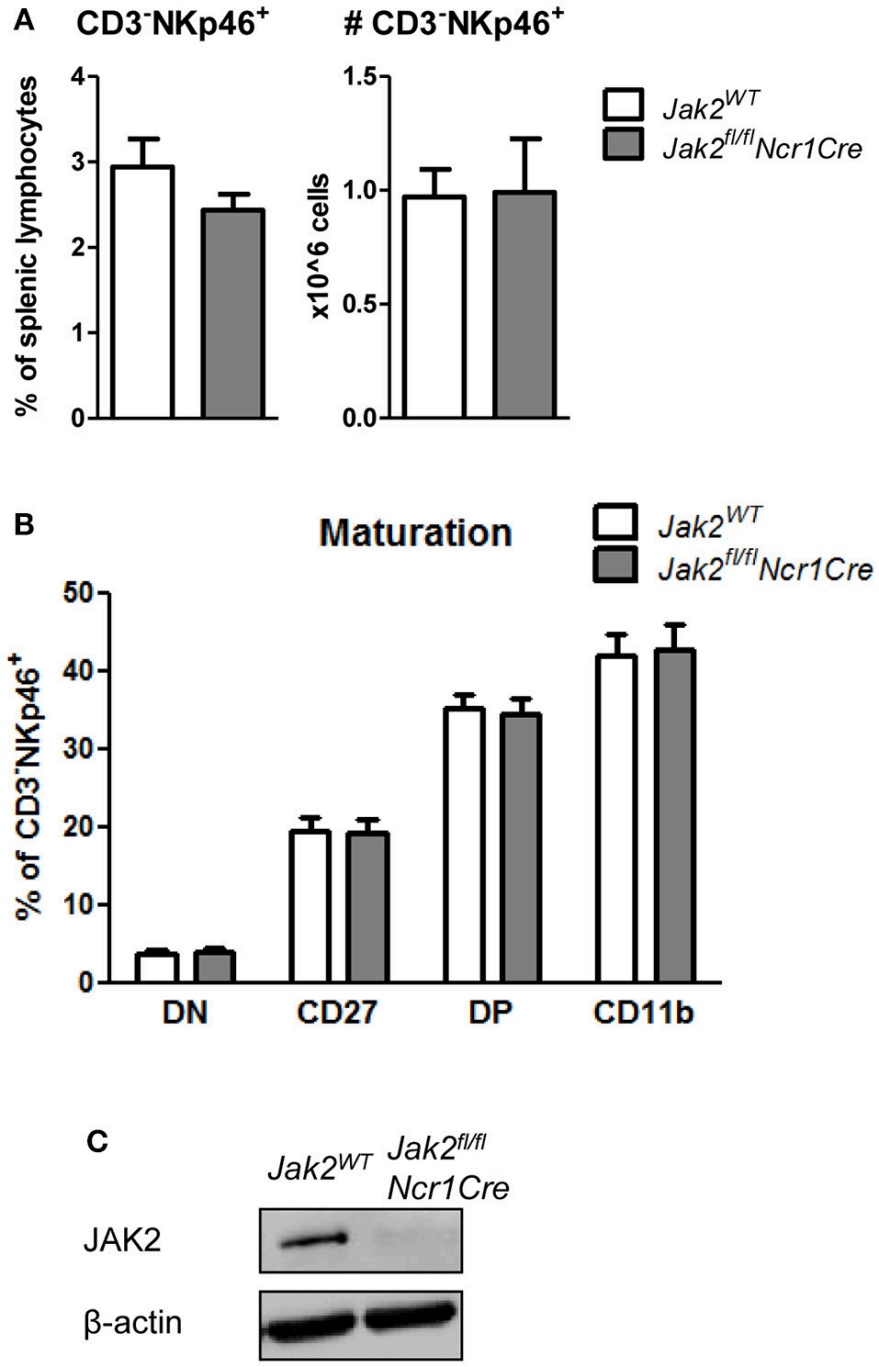
JAK2 is dispensable for NK cell survival and maturation. **(A)** Frequency (left panel) and total numbers (right panel) of CD3^−^NKp46^+^ NK cells in the spleens of *Jak2*^*WT*^ and *Jak2*^*fl*/*fl*^*Ncr1Cre* mice were assessed by flow cytometry. **(B)** Splenic CD3^−^NKp46^+^ NK cells were analyzed for expression of CD27 and CD11b by flow cytometry. Frequency of cells in each maturation stage is show: DN (CD27^−^CD11b^−^), CD27 (CD27^+^CD11b^−^), DP (CD27^+^CD11b^+^), CD11b (CD27^−^CD11b^+^). **(C)** The expression of JAK2 and β-actin was analyzed by Western blot in NK cells upon 6 days of expansion in IL-2. Scans of full blots are available in Supplementary Material. **(A,B)** Bar graphs represent mean ± SEM of 2–3 independent experiments; *n* = 5–12.

So far we showed that NK cell-intrinsic JAK1 deletion leads to NK cell deficiency. To elucidate if JAK2 impacts on NK cell survival and maturation, we analyzed splenic NK cells in *Jak2*^*fl*/*fl*^*Ncr1Cre* mice and their wild-type littermates. We failed to detect any impact of JAK2 deletion on the frequency or total numbers of CD3^−^NKp46^+^ cells in the spleen (Figure 3A). Furthermore, *Jak2*^*fl*/*fl*^*Ncr1Cre* mice showed normal NK cell maturation, as similar percentages of CD27^−^CD11b^+^ cells were detected in both genotypes (Figure 3B). As the deletion of JAK2 protein in NK cells was very efficient (Figure 3C), these data unequivocally define that unlike JAK1, NK cell-intrinsic JAK2 is dispensable for NK cell survival and maturation.

### Loss of One Allele of *Jak1* Impairs NK Cell Functionality

**Figure 4:**
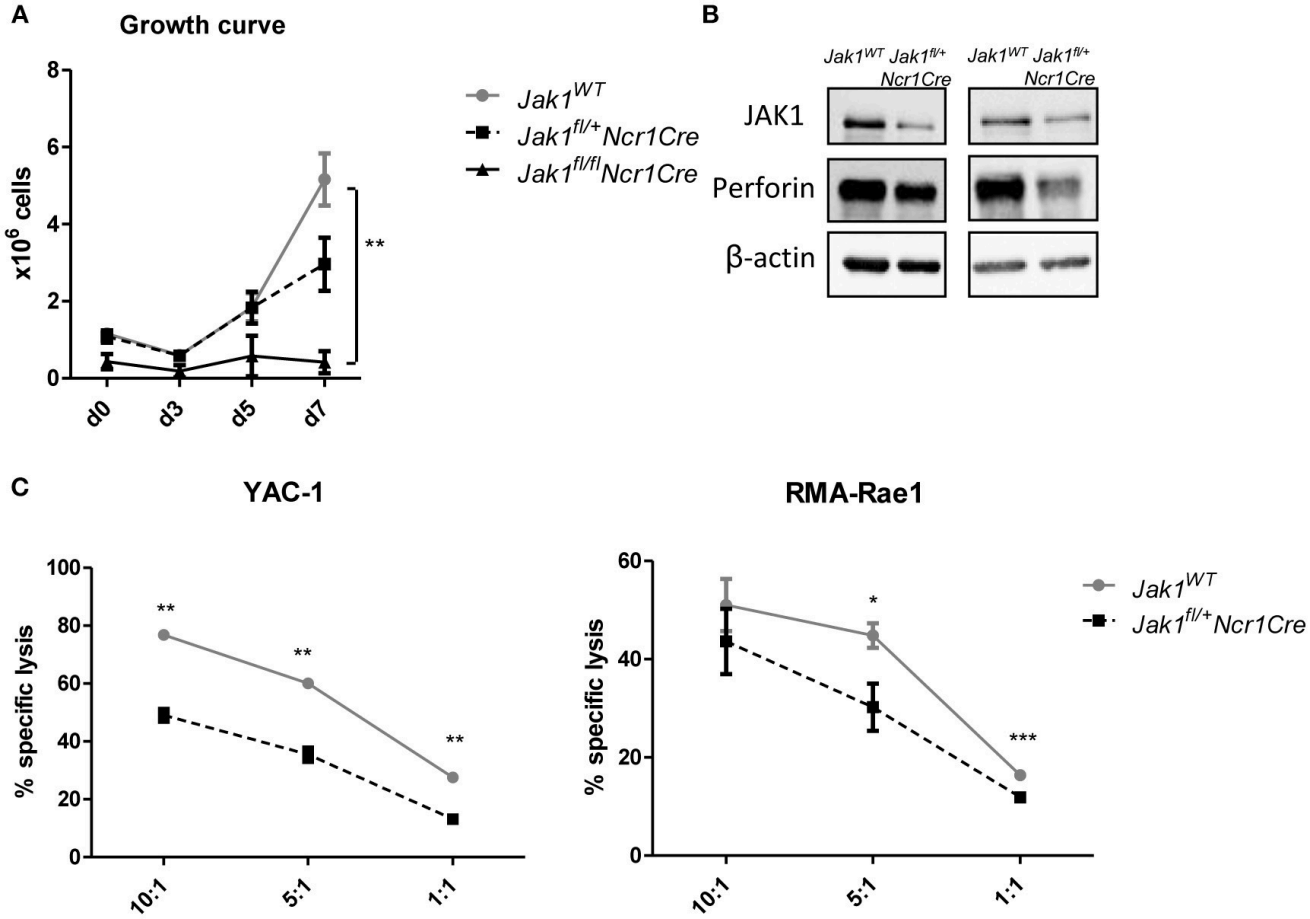
Loss of one allele of *Jak1* impairs NK cell activity**. (A)** NK cells were MACS-purified from spleens and cultured in IL-2 for 7 days. The numbers of living CD3^−^NK1.1^+^NKp46^+^ cells were assessed every 2–3 days. The symbols and the error bars present mean ± SEM of 2–4 biological replicates. **(B)** The expression of JAK1, Perforin and β-actin was analyzed in expanded NK cells from two independent experiments by Western blot. Scans of full blots are available in Supplementary Material. **(C)** Expanded NK cells from *Jak1*^*WT*^ and *Jak1*^*fl*/+^*Ncr1Cr* mice were mixed with CFSE-stained YAC-1 (left panel) or RMA-Rae1 (right panel) target cells at indicated effector target ratios. The specific lysis was assessed by flow cytometry. For YAC-1 a representative graph of one of two independent experiments is shown. The symbols and the error bars present mean ± SEM of 2 technical replicates. For RMA-Rae1 a mean out of two independent experiments is shown. The symbols and the error bars present mean ± SEM of 2–3 biological replicates. *p < 0.05, **p < 0.01, ***p < 0.001.

To get further insights into how JAK1 regulates NK cell functionality, we analyzed the growth of MACS-purified splenic NK cells from *Jak1*^*WT*^, *Jak1*^*fl*/+^*Ncr1Cre*, and *Jak1*^*fl*/*fl*^*Ncr1Cre*. JAK1-deficient NK cells did not expand, which shows that even under a very high dose of IL-2 other JAKs cannot compensate for the loss of JAK1 (Figure 4A). The loss of one allele of *Jak1* resulted in a minor growth deficiency (Figure 4A). Western blot analysis confirmed the reduced JAK1 protein expression in expanded *Jak1*^*fl*/+^*Ncr1Cre* NK cells which was paralleled by reduced levels of perforin (Figure 4B). In line, *Jak1*^*fl*/+^*Ncr1Cre* NK cells displayed an impaired cytotoxic activity against target cell lines YAC-1 and RMA-Rae1 (Figure 4C). These results prove that JAK1 is not only indispensable for maintaining NK cells in periphery, but also contributes to their cytotoxic activity.

### NK Cell Depletion Induced by Loss of One Allele of *Jak1* Impairs Tumor Surveillance

**Figure 5:**
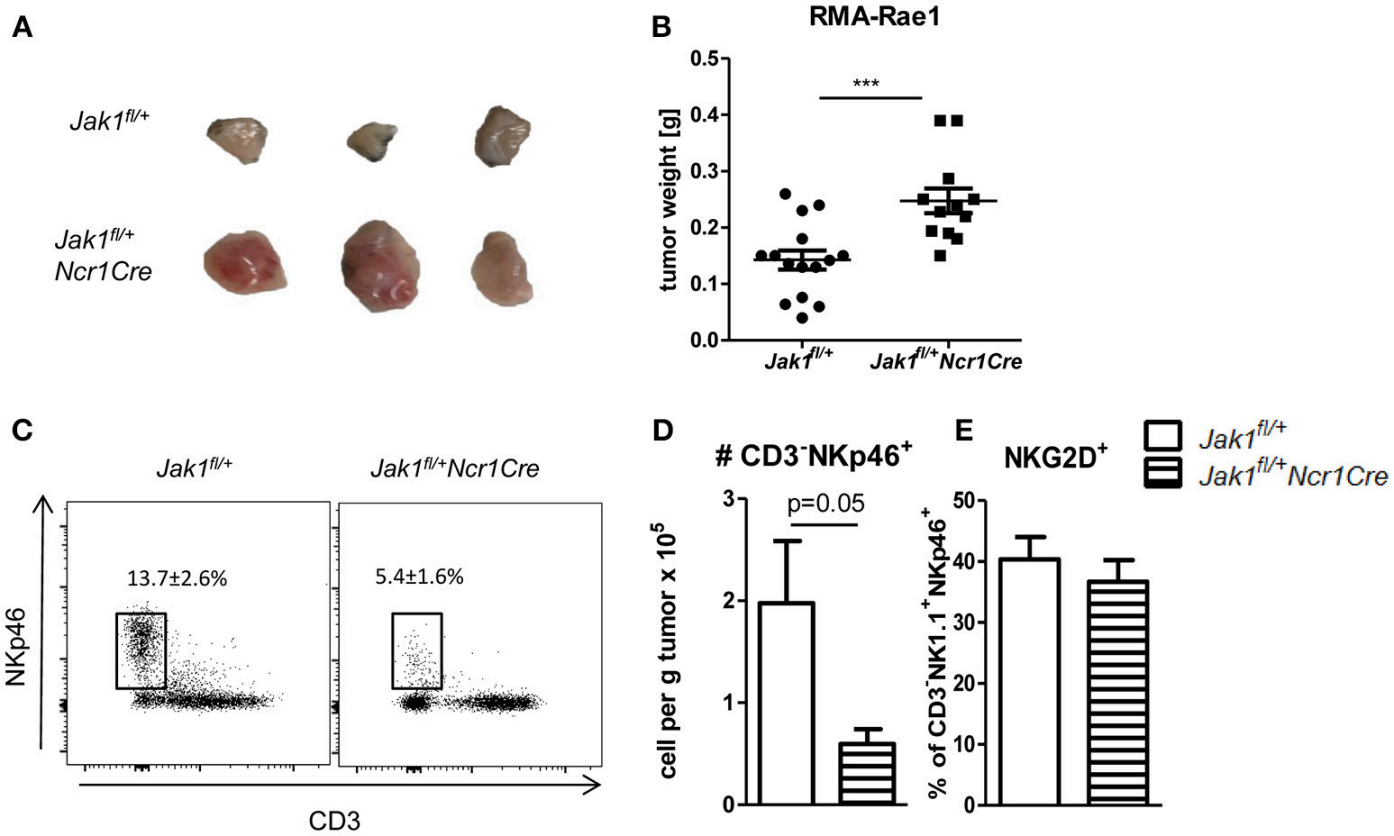
Loss of one allele of *Jak1* impairs tumor surveillance. **(A,B)** *Jak1*^*fl*/+^ and *Jak1*^*fl*/+^*Ncr1Cre* mice were injected *s.c*. with 10^6^ RMA-Rae1 cells and after 10 days the tumor weight was assessed. Shown are **(A)** representative tumor pictures and **(B)** dot plots with horizontal lines representing mean tumor weights ± SEM from 2 independent experiments; *n* = 6–8 **(C,D)** Tumor infiltrating NK cells were analyzed by flow cytometry. **(C)** Representative plots of tumor infiltrating CD3^−^NKp46^+^ cells and their percentage among Rae1^−^ cells are shown. **(D)** Total numbers of tumor infiltrating CD3^−^NKp46^+^ cells per gram of tumor and **(E)** percentages of NKG2D^+^ NK cells are presented as bar graphs showing mean ± SEM from one experiment *n* = 4–5. ***p < 0.001.

NK cells are crucial for the early recognition and elimination of transformed cells. To investigate whether NK cell reduction and their impaired functionality in *Jak1*^*fl*/+^*Ncr1Cre* mice results in increased susceptibility to tumor growth and is not compensated by other means, we made use of RMA-Rae1 lymphoma cells. This cell line is a tool to study NK cell-dependent tumor surveillance in a robust and efficient way (20). We subcutaneously transplanted RMA-Rae1 lymphoma cells into both flanks of *Jak1*^*fl*/+^ and *Jak1*^*fl*/+^*Ncr1Cre* mice. Lacking one allele of *Jak1* impaired the ability of NK cells to control the tumor growth as illustrated by increased tumor size (Figure 5A) and tumor weight in *Jak1*^*fl*/+^*Ncr1Cre* (Figure 5B). In line, these tumors showed significantly reduced NK cell infiltration (Figures 5C,D). We failed to detect any difference in the frequency of NKG2D^+^ tumor infiltrating NK cells (Figure 5E), which are crucial for the recognition of RMA-Rae1 tumors. In summary, our results show the reduced NK cell numbers combined with an impaired NK cell functionality in *Jak1*^*fl*/+^*Ncr1Cre* mice are sufficient to significantly impair tumor growth control *in vivo*.

## Discussion

Dysregulation of the JAK-STAT signaling pathway is tightly associated with cancer development as well as immune disorders (7). The first JAK-linked disease discovered was the severe combined immune deficiency (SCID) which was characterized by NK cell abnormalities caused by LOF JAK3 mutations (26, 27). We here show that deletion of JAK1 in NKp46^+^ cells leads to innate immune deficiency with loss of NK and ILC1 cells in peripheral organs, whereas JAK2 is redundant for NK cell survival and maturation.

Our previous work uncovered that loss of STAT5 in NK cells leads to severe reduction of NK cell numbers in peripheral organs (18). The loss of NK cells in *Stat5*^*fl*/*fl*^*Ncr1Cre* mice was rescued by enforced expression of the pro-survival molecule BCL2 (28). This study defined STAT5 as a crucial survival factor for NK cells. STAT5B-deficient mice largely lack NK cells (29), in line with the fact that STAT5 signals downstream of cytokines that are vital for NK cell biology, such as IL-2 or IL-15 (30). IL-15 is crucial for NK cell development and survival as *Il15*^−/−^ mice are largely devoid of peripheral NK cells (31). IL-15 signals via a receptor complex of γc receptor chain, IL-2Rβ, and IL-15rα (32). Knockout mice of each receptor chain prove an absolutely critical role for signals sent downstream of IL-15 for NK cell development (33, 34). To date, the contribution of γc-associated JAK3 to NK cell development has been well-established. Mice with JAK3 deficiency show a similar SCID phenotype as observed in human patients and NK cell development is blocked at the pre-NK progenitor stage (35, 36). Now we place previously underappreciated JAK1 as a crucial part of the IL-15/STAT5 axis in NK cells. *Jak1*^*fl*/*fl*^*Ncr1Cre* NK cells show a developmental block at the iNK cell stage and an almost complete loss of NK cells in peripheral organs.

Interestingly, the consequences of JAK1 deletion for NK cells exceed the effects of STAT5-deficiency. Impairment of combined STAT3 and STAT5 activation may underlie the more pronounced loss of NK cells, as STAT3 has been shown to induce expression of the crucial NK cell survival gene *Mcl1* (37, 38). Alternatively, JAK1 and STAT5 may have different half-lives which account for different frequencies of “just deleters”—NK cells which have just lost the gene but still carry the protein, that may explain differences.

The gene-dosage effect of JAK1-deficiency is reflected in NK cell numbers, while loss of one allele of JAK1 is dispensable for NK cell maturation. This suggests that activated STAT5 is rate limiting for NK cell survival but not maturation. Deletion of one allele of *Jak1* is also sufficient to significantly impair tumor surveillance based on decreased numbers of NK cells combined with a diminished functionality of the remaining NK cells. In accordance with our data, NK cell-specific deletion of the STAT5 target gene *Mcl1* leads to severe NK cell deficiency which causes a significant increase in metastatic burden (38).

JAKs may exhibit redundant functions and compensate for each other. Downstream of interferon γ receptor JAK1 can partially compensate for loss of JAK2 kinase activity (39). JAK2 has also been shown to phosphorylate STAT5 downstream of IL-15 during *in vitro* differentiation of NK cells (16). On the other hand, constitutively active JAK2 can only modestly compensate for the loss of JAK1 in stem cells suggesting a non-redundant role of JAK1 and 2 in HSCs. In line, we observe that in the absence of JAK1, JAK2 fails to compensate in activating STAT5, even under high dose of IL-2 *in vitro*, to allow NK cell survival. It remains to be elucidated whether compensatory effects are achievable by expressing a constitutively active form of JAK2. More importantly, we show that loss of JAK2 in NKp46^+^ cells is dispensable for NK cell survival. JAK2-deficient NK cells are fully mature, proving that NK cell-intrinsic JAK2 is not driving NK cell maturation. This also indicates that the impaired NK cell maturation in *Jak2*^*fl*/*fl*^*Mx1Cre* mice (14) is most likely caused by NK cell-extrinsic functions of JAK2. One might speculate that the constitutive deletion of JAK2 alters the cytokine milieu.

Our data provide novel insights into results obtained upon JAK1/JAK2 inhibitor treatment. Inhibition of JAK1/JAK2 reduces NK cell numbers and maturation (14, 15), which according to our data is clearly the effect of inhibiting JAK1 rather than JAK2. We thus propose that the development of JAK2-specific inhibitors may be advantageous as they would leave NK cell-mediated tumor surveillance intact. This might be of particular relevance in the case of JAK2-driven leukemia such as JAK2^V617F^-induced myeloproliferative neoplasms.

## Author Contributions

AW-S, KK, DP, and NL performed experiments. AW-S analyzed the data. VS, GS, and AD provided the resources. AW-S and VS wrote the manuscript.

### Conflict of Interest Statement

The authors declare that the research was conducted in the absence of any commercial or financial relationships that could be construed as a potential conflict of interest.

## Abbreviations

FACS: fluorescence-activated cell sorting
HSCs: hematopoietic stem cells
ILC: innate lymphoid cells
JAK: Janus kinase
LOF: loss of function
NK: natural killer
PID: primary immunodeficiency
SCID: severe combined immune deficiency
STAT: signal transducer and activator of transcription.
