# Supplementary material for: Loss of JAK1 Drives Innate Immune Deficiency
Source: Front Immunol. 2019 Jan 8;9:3108. doi: 10.3389/fimmu.2018.03108 (PMC6331462; doi:10.3389/fimmu.2018.03108)
Supplement: Supplementary file 1 [file Data_Sheet_1.PDF]

Figure S1

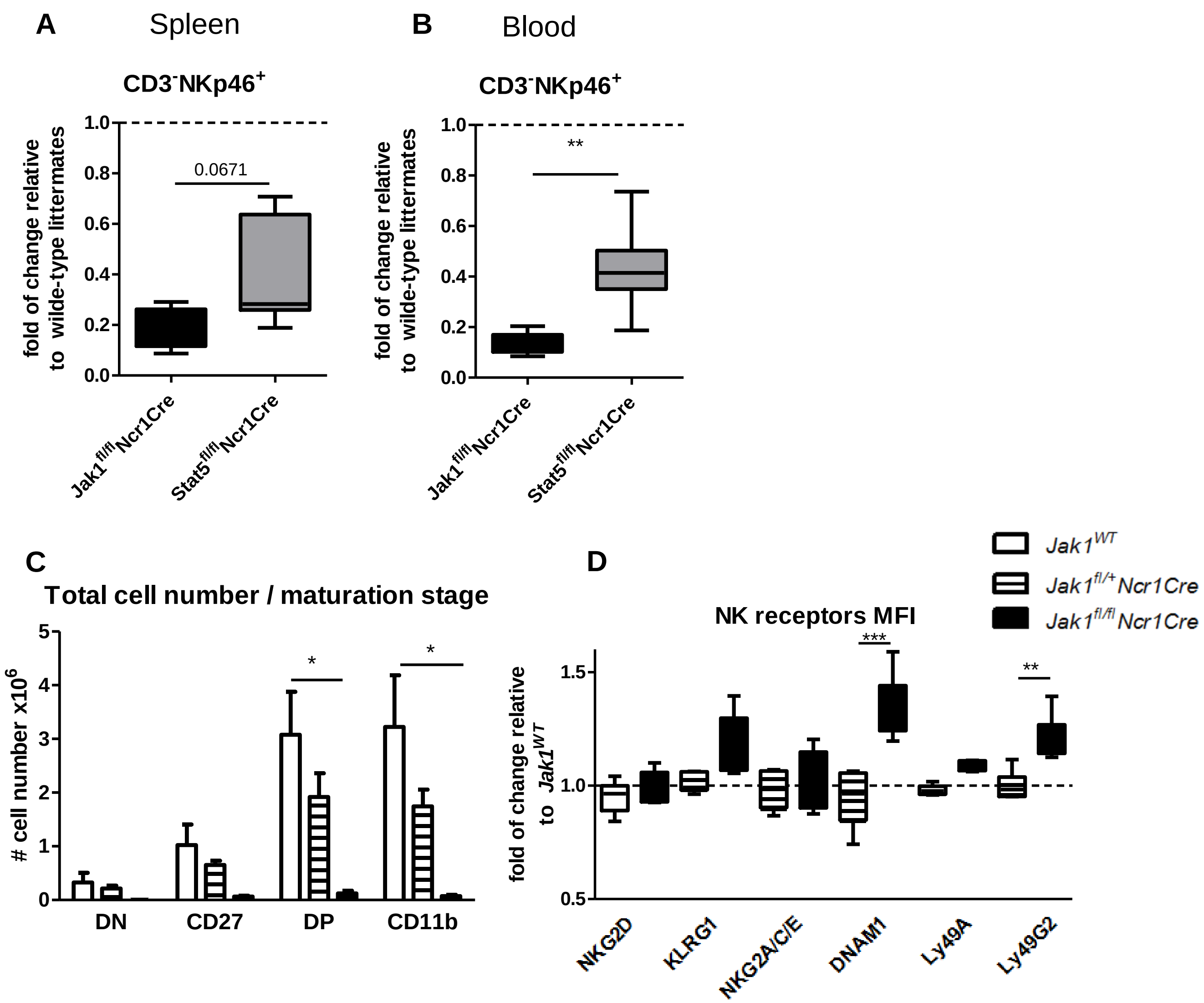

Figure S1. JAK1-deficiency results in more severe loss of NK cells than deletion of STAT5. (A, B) Fold of decrease of NK cell percentage in *Jak1*$^{fl/fl}$*Ncr1Cre* and *Stat5*$^{fl/fl}$*Ncr1Cre* compared to wild-type littermates which are marked by dotted line. The CD3$^-$NKp46$^+$ cells were analyzed by flow cytometry in the (A) spleen and (B) blood. (C) The total number of CD3$^-$NKp46$^+$ cells in each maturation stage (DN (CD27$^-$CD11b$^-$), CD27 (CD27$^+$CD11b$^-$), DP (CD27$^+$CD11b$^+$), CD11b (CD27$^-$CD11b$^+$) was assessed by flow cytometry. (D) The expression of NKG2D, KLRG1, NKG2A/C/E, DNAM1, Ly49A or Ly49G2 in splenic CD3$^-$NKp46$^+$ cells was analyzed by quantifying the median fluorescence intensity (MFI) of receptor positive population. The MFI of each receptor in *Jak1*$^{fl/+}$*Ncr1Cre* and *Jak1*$^{fl/fl}$*Ncr1Cre* mice is shown as fold of change relative to *Jak1*^{WT}. (A-D) Boxes with whiskers or bar graphs represent mean ± SEM of 1-2 independent experiments; n=4–7.
