# Supplementary material for: Loss of JAK1 Drives Innate Immune Deficiency
Source: Front Immunol. 2019 Jan 8;9:3108. doi: 10.3389/fimmu.2018.03108 (PMC6331462; doi:10.3389/fimmu.2018.03108)

Supplementary information: Scan of full western blot from Fig. 3C

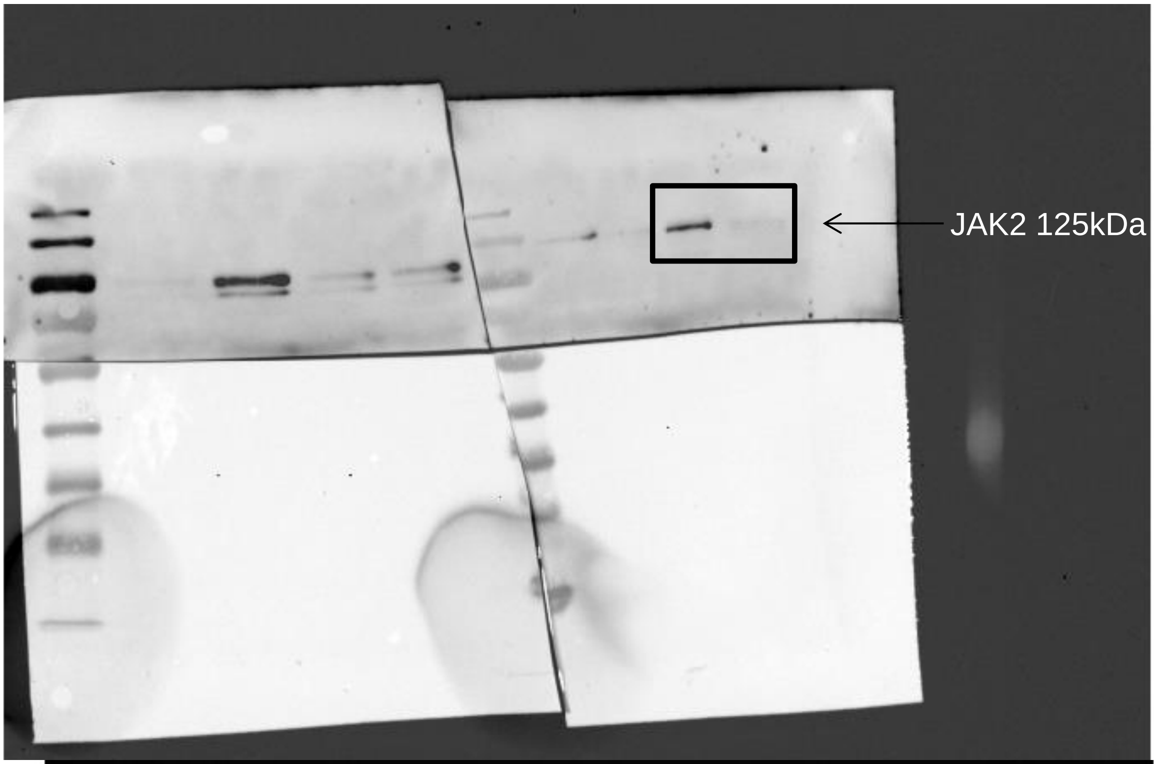

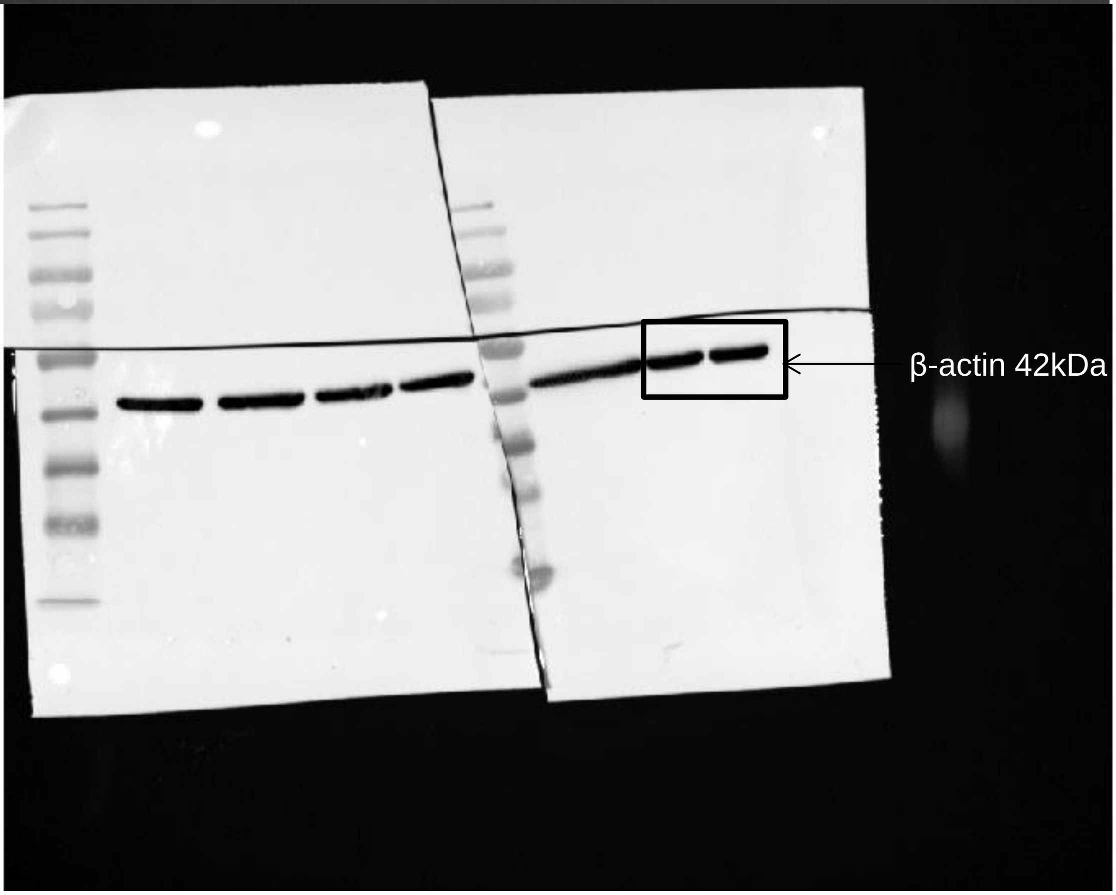

Supplementary information: Scan of full western blots from Fig. 4B

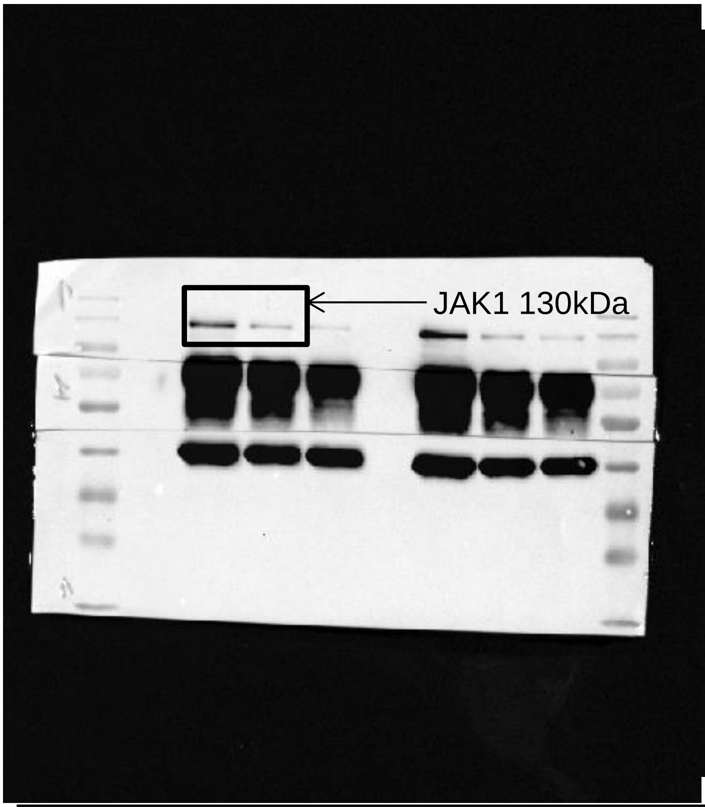

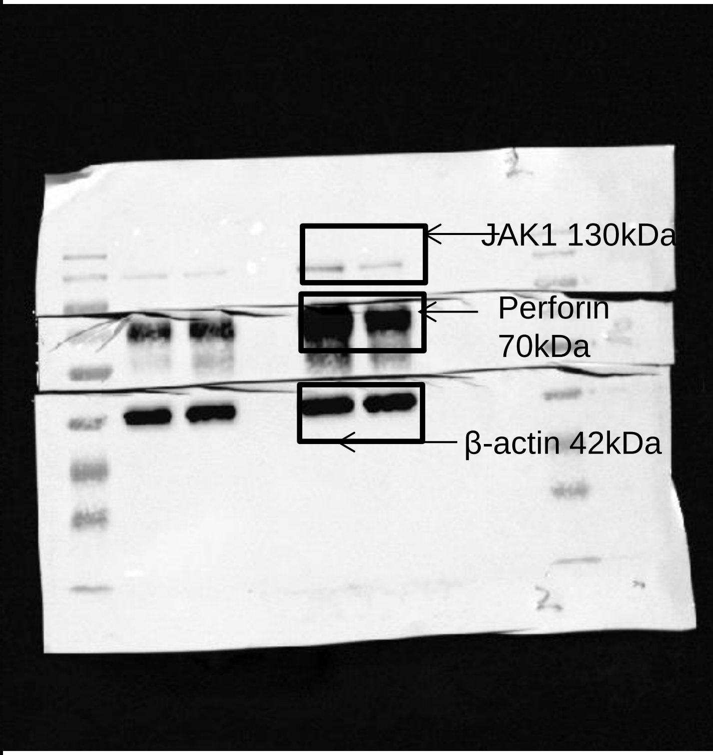

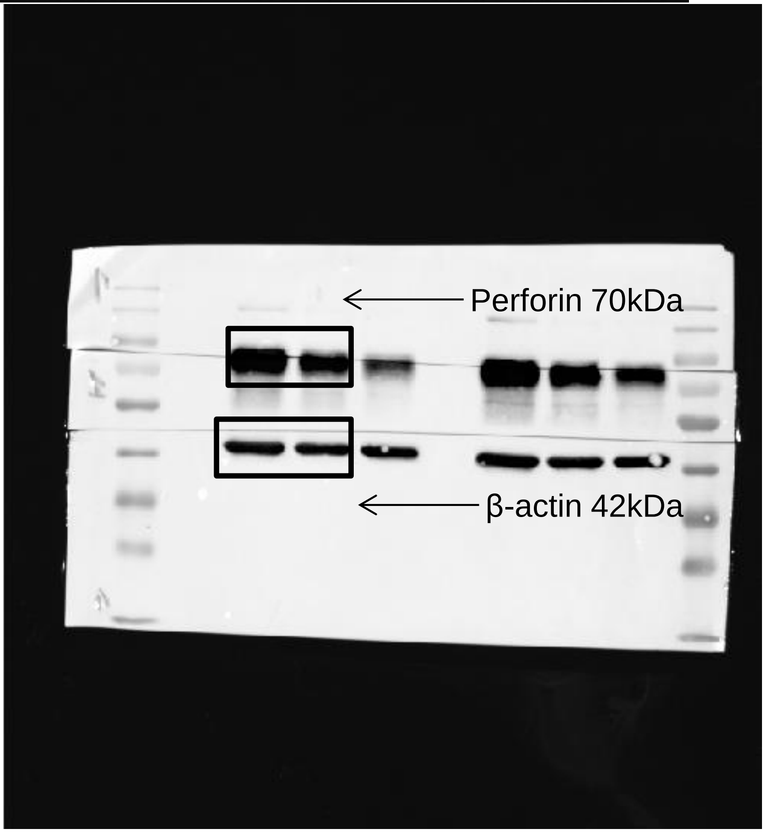

Supplement: Supplementary file 2 [file Data_Sheet_2.PDF]
